# Partial Replacement of Peat: Effects on Substrate Physico-Hydrological Properties and Sage Growth

**DOI:** 10.3390/plants14172801

**Published:** 2025-09-07

**Authors:** Anna Elisa Sdao, Sonia Cacini, Danilo Loconsole, Giulia Conversa, Giuseppe Cristiano, Antonio Elia, Barbara De Lucia

**Affiliations:** 1 Department of Soil, Plant and Food Sciences, University of Bari Aldo Moro, Via Amendola 165/A, 70126 Bari, Italy; anna.sdao@uniba.it (A.E.S.); danilo.loconsole@uniba.it (D.L.); giuseppe.cristiano@uniba.it (G.C.); 2 CREA Research Centre for Vegetables and Ornamental Crops, Council for Agricultural Research and Economics, Via dei Fiori 8, 51017 Pescia, Italy; 3 Department of Agriculture, Food, Natural Resources and Engineering (DAFNE), University of Foggia, via Napoli 25, 71100 Foggia, Italy; giulia.conversa@unifg.it (G.C.); antonio.elia@unifg.it (A.E.)

**Keywords:** brewer’s spent grain, by-product valorization, circular alternative, coffee silverskin, potted ornamentals, soilless cultivation, sustainable growing medium, wood fiber

## Abstract

The transformation of organic by-products derived from waste into value-added resources represents a promising strategy to advance circular economy principles and bolster environmental and agricultural sustainability, especially in soilless cultivation. This study evaluates the viability of three organic by-products—wood fiber (WF), coffee silverskin (CS), and brewer’s spent grains (BSGs)—as partial peat replacements in horticultural substrates. Ten growing media formulations were assessed, incorporating increased doses (0–40% *v*/*v* as peat replacement-PR) of each alternative by-product. The effects on physical and hydraulic substrate properties, along with plant growth traits, were examined using two ornamental *Salvia* genotypes, ‘Victoria’ and ‘Amistad’. To synthesize the multivariate growth data into a single, biologically meaningful metric, based on the first principal component, a Growth Index (GI), a PC1-derived index, was calculated, providing a powerful, unified metric to rank substrate efficacy. WF-based substrates exhibited increased porosity and diminished water retention, whereas media enriched with CS and BSG enhanced moisture availability, particularly at 20–40 PR. The bulk density was highest at PR40 for both WF and BSG treatments, and at PR20 in CS-based substrates. Electrical conductivity increased in CS and BSG treatments with rising PR levels. The results on the vegetative growth of ornamental sages have highlighted that differential PR rates are required depending on the specific organic by-product and plant genotype. In ‘Victoria’, GI indicates that a 20% replacement of peat with BSG provided the optimal conditions for holistic plant development; the lowest GI for WF substrates across nearly all peat replacement levels indicated that it was the most detrimental alternative for this cultivar. In ‘Amistad’, the analysis of the GI scores revealed that the CS20 and BSG20 of peat replacement yielded the highest overall growth, with GI scores significantly greater than those of the peat control. CS10 and BSG40 also showed high GI scores in ‘Amistad’. WF10 had GI scores similar to those of the peat control. In general, the GI-based approach confirms that moderate inclusion of brewer’s spent grain (BSG20) is a highly effective peat replacement for both genotypes. At the same time, coffee silverskin (CS) is particularly effective for the ‘Amistad’ genotype. This analysis underscores that optimal substrate formulation is not only dependent on the amendment type and rate but also critically on the plant genotype.

## 1. Introduction

The ornamental industry produces potted plants, grown in soilless cultivation, for their esthetic value from a wide range of species [1], which are distinguished by broad agronomic practices with specific requirements in terms of substrate. This is also referred to as growing medium, which consists of solid and porous materials of natural or synthetic origin, mineral or organic. They form the structural and functional basis for root development [2]. Schindler et al. [3] defined the growing medium as a man-made environment designed to optimize water and nutrient availability, provide mechanical support, and promote microbial interactions essential for plant growth and productivity. To fulfill these roles effectively, substrates must satisfy several key requirements. Physically, it should have a porous and stable structure to ensure adequate aeration and water-holding capacity [4,5,6]. Chemically, it must offer consistency, maintaining stable chemical properties over time and be free of toxic compounds [7]. Biologically, it should support beneficial microorganisms and suppress pathogens [8]. In addition, practical attributes such as lightweight, affordability, ease of handling and local availability are equally critical for sustainable production [9,10]. Historically, peat has served as the primary component of commercial horticultural substrates due to its superior moisture retention, structural stability, and compatibility with a wide range of crops [11,12]. Global peat extraction is estimated to be approximately 90 million m^3^ annually, with nearly 40 million m^3^ allocated to horticulture. In Europe alone, approximately 20 million tons are harvested annually [13,14]. However, growing environmental concerns, such as greenhouse gas emissions and the destruction of wetland ecosystems, have prompted regulatory action [15,16]. Countries such as the United Kingdom have announced bans on peat use in amateur gardening by 2026 and in the professional horticulture sector by 2030 [17,18]. Despite these policy efforts, the transition away from peat remains technically challenging. Commercial growers require alternative substrates that can match the performance of peat across multiple agronomic parameters without compromising crop yield or quality. In this context, one promising strategy involves the valorization of organic by-products generated by the forestry and agri-food sectors [19]. These materials, often produced in large volumes and locally available, align well with circular economy principles and offer significant potential as sustainable substrate components [20,21].

### 1.1. Wood Fiber

Global demand for wood fiber (WF) is projected to increase by approximately 1000% by 2050, accompanied by an estimated requirement of 65 Mm^3^ year^−1^ of novel materials to support the expanding adoption of soilless cultivation systems worldwide [13]. WF, a by-product of wood processing, is a growing material in the substrate industry, although it is usually not more than 30% (*v*/*v*) with peat [22]. As a renewable and biodegradable resource derived from products of wood manufacture, it exhibits several favorable properties for use in soilless cultivation. It has a low bulk density, good drainability and wettability, and can optimize the physical properties and reduce the weight of the growing media [7,23]. WF is a stable, pathogen-free material that could help reduce dependence on non-renewable substrates [24]. However, the increasing need for biomass for energy and heat production presents significant competition for the substrate sector in securing access to this raw material [25].

### 1.2. Coffee Silverskin

Coffee silverskin (CS), which represents 4.2% of coffee green beans’ total weight, is a by-product of coffee roasting and is currently discarded as waste, but exhibits several favorable properties for substrate utilization. It is lightweight, biodegradable, pathogen-free, and renewable, and may contribute functional compounds, such as antioxidants, to the growing medium [21,26].

### 1.3. Brewer’s Spent Grain

Similarly, brewer’s spent grain (BSG), which accounts for up to 85% of brewery by-product, is another abundant organic residue [27]; it consists of layers of peel, pericarp, and seeds with residual amounts of endosperm and aleurone from barley. With global production estimated at 37.2 million tons in 2021—approximately 21–22 kg generated per 100 L of beer—BSG is microbiologically sterile post-production due to thermal processing. However, its high moisture and nutrient content make it highly perishable [28,29]. When stabilized and incorporated into substrates in small proportions (<5%), BSG may act as a biofertilizer and enhance the organic content of the medium [30].

### 1.4. Hydrophysical Recommended Ranges

The EU is promoting research on sustainable practices to recover high-quality resources from organic residues and by-products, aligning with the Green Deal objectives [31], the Farm to Fork (F2F) Strategy [32] and the new Circular Economy Action Plan [33]. The effectiveness of any growing medium, including those incorporating organic by-products, is closely linked to its physical and hydraulic characteristics, particularly the balance between water availability and air-filled porosity. These properties are primarily influenced by the material composition, particle size distribution, and container geometry [34,35,36].

The recommended ranges for the commercial production of soilless crops are as follows: dry bulk density (BD) < 0.4 g cm^−3^, total porosity space (TPS) > 85%, air content (AC) 20–30% vol., total water-holding capacity (WHC) 550–700 mL L^−1^ and a water volume content at −1 kPa in the range 55–70% vol. [37]. Maintaining substrates within the recommended physical property ranges [38] is expected to reduce the sensitivity of plant production management; however, outcomes may still vary depending on the specific cultivation practices of the growers. Assessing the similarities and differences in hydrophysical properties between forestry and beverage by-products, as well as conventional components such as peat, constitutes a crucial step in enhancing their potential as components of potting media.

### 1.5. Ornamental Sages

*Salvia* is the largest genus in the Lamiaceae family, with 1036 accepted species to date [39], widely cultivated for its ornamental, medicinal, and aromatic properties [40,41], and constitutes almost one-quarter of this family [42]. It is widely distributed in temperate, subtropical, and tropical regions worldwide [43]. *Salvia* spp. are also important ornamental plants due to their beautiful leaves, inflorescence variations, and hardiness in dry and hot [44,45] or wet [46] and cold [47] environments. Ornamental sages are propagated asexually both in vivo [48,49] and in vitro [50]. The floriculture industry continuously strives to introduce new cultivars into the market. In this context, cultivars and interspecific hybrids among *Salvia* species offer the potential to exploit the extensive variability in leaf morphology, fragrance, flower color, and inflorescence architecture, as well as for the agronomic needs of cultivation and planting in green spaces.

Despite the great importance of partial peat replacement in growing media, in recent years, few studies have investigated the species and cultivars of *Salvia*. Greco et al. [51] in potted *S. officinalis* L., experimented with the use of vermicompost, compost, and solid digestate as valid peat alternatives. In *S. rosmarinus* Spenn., the application of 10% smoked biochar displayed a subtle stimulating effect compared to plants grown in substrates composed solely of 100% peat [52].

### 1.6. Aim of Research

Many studies have focused on the role of physico-hydraulic properties of substrates with reduced peat content in influencing the agronomic performance of ornamental plants [53,54,55,56,57]. Additionally, other studies have examined physical characteristics [58,59,60] to assess the feasibility of successfully replacing peat in ornamental plant production.

Building on this background, the present study evaluated the feasibility of incorporating forestry and drink industry by-products—WF, CS, and BSG—as partial peat replacement (PR) at increasing doses, monitoring the growth of potted ornamental sages. The focus was on assessing their physico-hydraulic behavior and crop growth in the context of a circular, sustainable and high-performance greenhouse pot-based growing system.

## 2. Results

### 2.1. Physical and Hydrological Characterization

Table 1 presents the ANOVA results for the effects of peat replacement (PR) with wood fiber (WF), coffee silverskin (CS), and brewer’s spent grain (BSG) on physical properties of the substrates assessed before cultivation.

**Figure 1:**
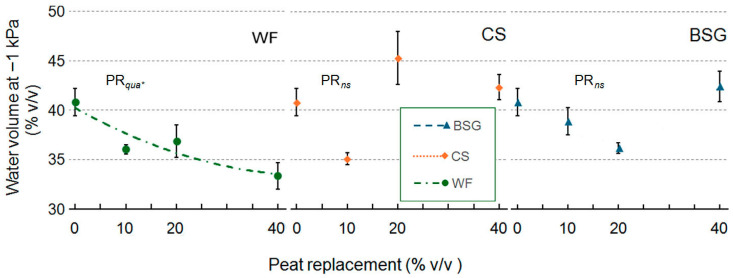
Effect of peat replacement level (PR, 0–40% *v*/*v*) with three by-product materials—wood fiber (WF), coffee silverskin (CS), or brewer’s spent grain (BSG)—on water volume at −1 kPa. Data points represent means ± standard error (n = 3). Statistical analysis performed through ANOVA: ns and *, not significant or significant at *p*≤ 0.05, respectively. Orthogonal polynomial contrasts were employed to evaluate quadratic (*qua*) trend.

Water volume at −1 kPa (WV) (Figure 1) was affected differently by peat replacement (PR) matrices: both wood fiber (WF) and brewer’s spent grain (BSG) substrates exhibited an asymptotic approach; coffee silverskin (CS) did not significantly alter WV across the tested range. When WF replaces peat at 20%, as expected, the WV reaches a lower value than that of the 0PR. With BSG, at 0 and 40% PR, the WV values appear comparable, therefore difficult to interpret

**Figure 2:**
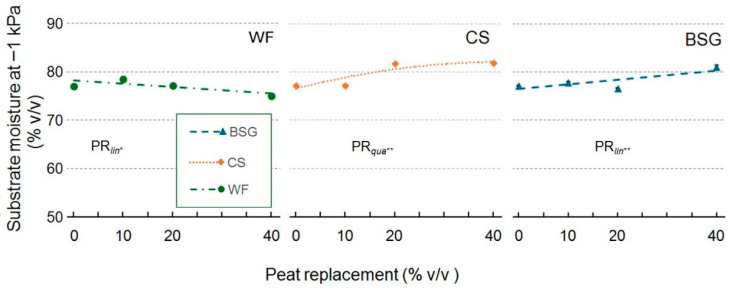
Effect of peat replacement level (PR, 0–40% *v*/*v*) with three by-product materials—wood fiber (WF), coffee silverskin (CS), or brewer’s spent grain (BSG)—on substrate moisture at −1 kPa. Data points represent means ± standard error (n = 3). Statistical analysis performed through ANOVA: * and **, significant at *p* ≤ 0.05 or *p* ≤ 0.01, respectively. Orthogonal polynomial contrasts were employed to evaluate linear (*lin*) and quadratic (*qua*) trends.

Substrate moisture (SM) at −1 kPa, which is a parameter close to field capacity (Figure 2) when BSG was used as the substrate for PR, was not significant. In contrast, with WF, values decreased as replacement levels increased. With CS as PR, SM increased quadratically, reaching its most significant peak at doses between 20 and 40% PR.

**Figure 3:**
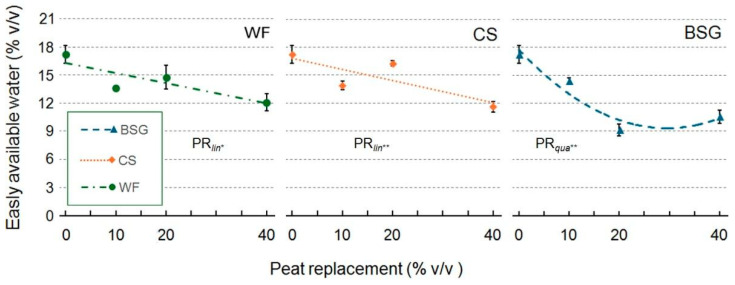
Effect of peat replacement level (PR, 0–40% *v*/*v*) with three by-product materials—wood fiber (WF), coffee silverskin (CS), or brewer’s spent grain (BSG)—on easily available water. Data points represent means ± standard error (n = 3). Statistical analysis performed through ANOVA: * and **, significant at *p* ≤ 0.05 or *p* ≤ 0.01, respectively. Orthogonal polynomial contrasts were employed to evaluate linear (*lin*) and quadratic (*qua*) trends.

PR generally reduced the amount of easily available water (EAW) (Figure 3), although the effect varied depending on the substrate type. With WF and CS as PR, easily available water decreased linearly as the percentage of peat replacement increased. In contrast, the use of BSG resulted in a distinct non-linear, quadratic response. The relationship for BSG was convex, with EAW declining to a minimum at approximately 20% PR. Crucially, beyond this point, the EAW values appeared to stabilize, suggesting that a plateau was reached rather than a continued decline. This indicates that BSG’s impact on water availability is limited beyond a certain application rate.

**Figure 4:**
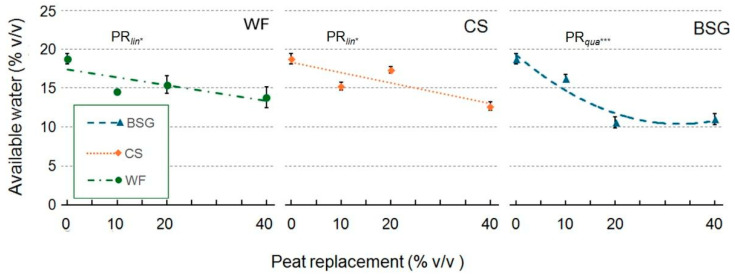
Effect of peat replacement level (PR, 0–40% *v*/*v*) with three by-product materials—wood fiber (WF), coffee silverskin (CS), or brewer’s spent grain (BSG)—on available water. Data points represent means ± standard error (n = 3). Statistical analysis performed through ANOVA: * and ***, significant at *p* ≤ 0.05 or *p* ≤ 0.001, respectively. Orthogonal polynomial contrasts were employed to evaluate linear (*lin*) and quadratic (*qua*) trends.

The available water results (Figure 4) followed the same trend as those relating to EAW.

**Figure 5:**
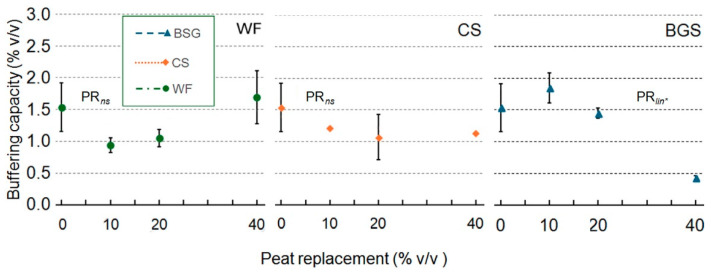
Effect of peat replacement level (PR, 0–40% *v*/*v*) with three by-product materials—wood fiber (WF), coffee silverskin (CS), or brewer’s spent grain (BSG)—on buffering capacity. Data points represent means ± standard error (n = 3). Statistical analysis performed through ANOVA: ns and *, not significant or significant at *p* ≤ 0.05, respectively. Orthogonal polynomial contrasts were employed to evaluate linear (*lin*) trend.

The water buffering capacity (BC) (Figure 5) was not significantly affected when WF and CS replaced the peat. However, when using BSG as a substrate, the BC exhibited a negative response as the PR level increased. Trend lines are shown for consistency and to aid in the visual interpretation of potential linear or quadratic trends, even where ANOVA results indicate non-significance (WF and CS).

**Figure 6:**
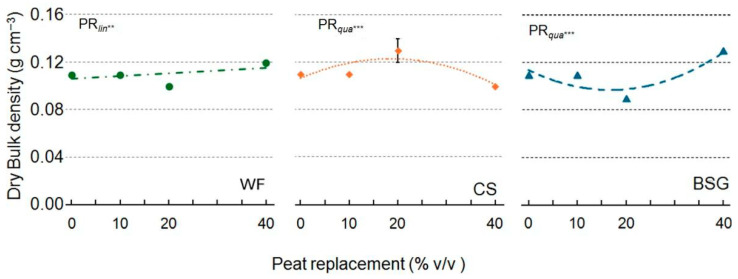
Effect of peat replacement level (PR, 0–40% *v*/*v*) with three by-product materials—wood fiber (WF), coffee silverskin (CS), or brewer’s spent grain (BSG)—on bulk density. Data points represent means ± standard error (n = 3). Statistical analysis performed through ANOVA: ** and ***, significant at *p* ≤ 0.01, or *p* ≤ 0.001, respectively. Orthogonal polynomial contrasts were employed to evaluate linear (*lin*) and quadratic (*qua*) trends.

When WF was used as the substrate, the dry bulk density (BD) (Figure 6) showed a linear increase, reaching the highest value at the 40% PR level. In contrast, with CS and BSG as substrates, a quadratic response with a concave course in the CS and a convex course in the BSG was observed. The reverse trend is apparent in the air spaces, so this is an effect of compaction driven by the 20% replacement pots.

**Figure 7:**
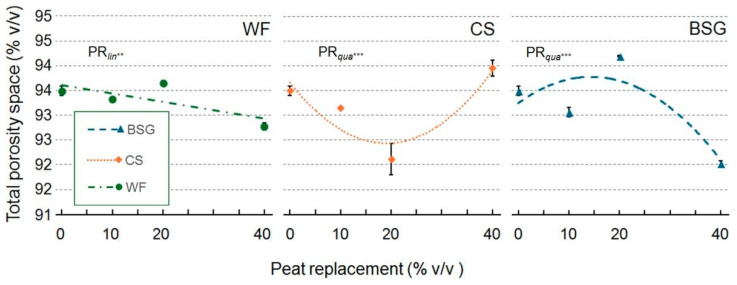
Effect of peat replacement level (PR, 0–40% *v*/*v*) with three by-product materials—wood fiber (WF), coffee silverskin (CS), or brewer’s spent grain (BSG)—on total porosity. Data points represent means ± standard error (n = 3). Statistical analysis performed through ANOVA: ** and ***, significant at *p* ≤ 0.01, or *p* ≤ 0.001, respectively. Orthogonal polynomial contrasts were employed to evaluate linear (*lin*) and quadratic (*qua*) trends.

Wood fiber as a substrate reduced the total porosity space (TPS) linearly as the PR percentage increased (Figure 7). With CS and BSG substrates, this property exhibited an asymptotic trend, convex in the CS and concave in the BSG.

### 2.2. Volumetric Water Content

**Figure 8:**
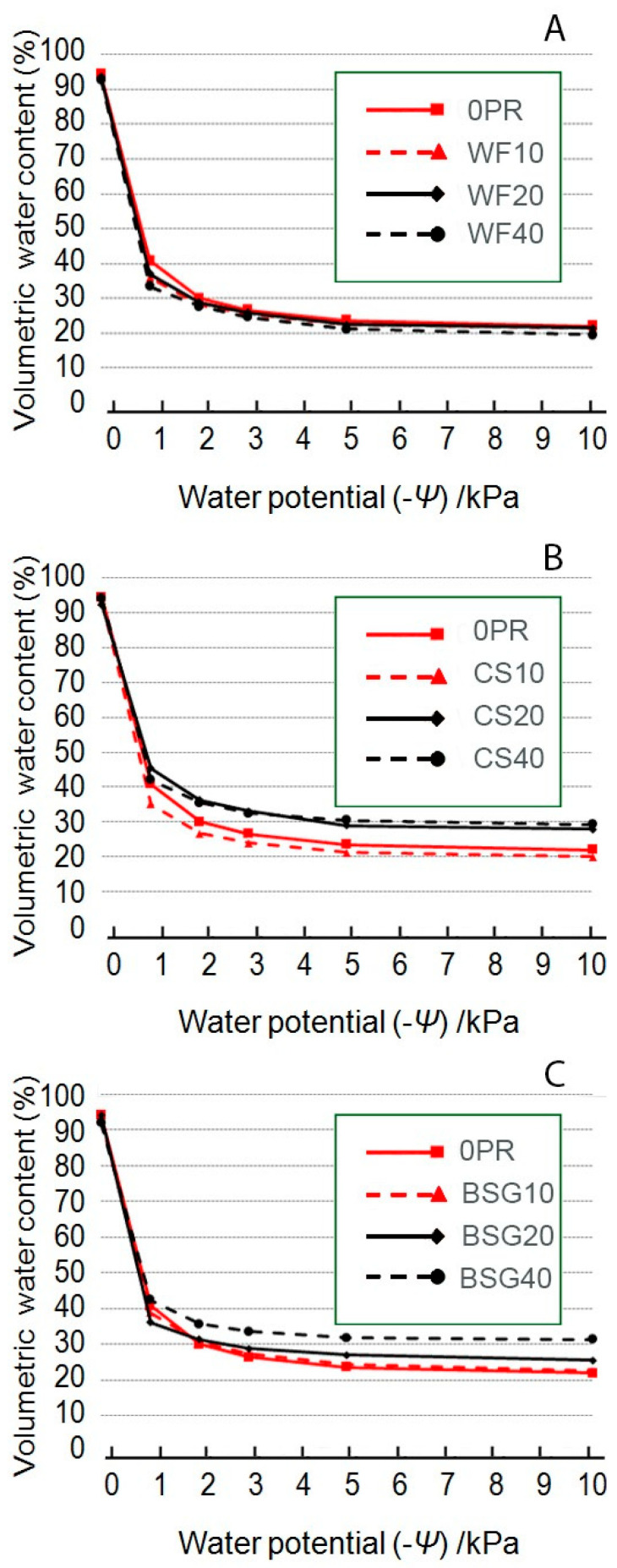
Effect of partial peat replacement (PR at 0, 10, 20 and 40% levels *v*/*v*) with wood fiber (WF, (**A**)), coffee silverskin (CS, (**B**)), or brewer’s spent grain (BSG, (**C**)) on the water retention curve. Each water retention curve was determined on the basis of 3 replicates.

Volumetric water content (VWC) shows the behavior of the peat-based substrate at increasing percentages of WF (Figure 8A), CS (Figure 8B) and BSG (Figure 8C).

Figure 8A presents the VWC across varying matric suctions (0 to −10 kPa) for peat control (0PR) and wood fiber-based substrates (10, 20 and PR40). The water retention curves shows clear and gradual shifts in hydraulic behavior as the proportion of WF increases, with significant implications for substrate water availability. At 0 kPa, all substrates displayed high VWC values, with a gradual decline from 0PR (94.3%) to WF40 (92.8%). Substrates did not differ significantly in Air Content (AC) at −1 kPa, which ranged from 44.9% to 59.5%, indicating a similar amount of gravitational water regardless of WF content. Conversely, the Total Available Water (TAW) decreased significantly, with the highest value (18.8%) recorded for 0PR and the lowest (13.8%) for WF40.

Figure 8B shows the water retention curves for the 0PR and CS substrates with PR of 10, 20, and 40% (*v*/*v*). The water retention curves show distinct changes in substrate hydrophysical behavior as CS content increases, suggesting a potential impact on water availability and substrate performance. Under saturated conditions, all substrates exhibited high VWC, with the 0PR showing the highest value (94.3%), followed by CS40 (93.9%), CS10 (93.1%), and CS20 (92.1%).

Differences became more evident as matric suction increased with a substantial reduction in VWC at −1 kPa observed across all treatments. The 0PR retained 40.8% VWC, while CS40 showed a more pronounced decline to 33.1%, highlighting reduced water retention with a higher CS level. Intermediate treatments (CS10 and CS20) exhibited moderate VWC losses (38.4 and 36.1%, respectively), consistent with their CS proportions. As tension further increased to −2 and −3 kPa, the VWC continued to decrease, with CS-based substrates consistently retaining less water than the peat control. By −5 kPa tension, the gap among treatments narrowed slightly but remained relevant. At −10 kPa, all substrates had reached the hygroscopic water range, yet differences persisted: 0PR retained the highest residual water content (22.1%), while CS40 showed the lowest (18.9%).

Figure 8C shows the water retention curves for 0PR and brewer’s spent grain-based substrates (BSG 10, 20 and 40% PR). At 0 kPa tension, all substrates demonstrated high VWC under saturated conditions, with the highest value recorded in 0PR at 94.3%, followed by BSG40 (93.9%), BSG10 (93.1%), and BSG20 (92.1%), indicating a slight initial decrease in VWC with increasing BSG percentage. At tension of −1 kPa, a sharp drop in VWC occurred across all substrates: 0PR maintained the highest value (40.8%), whereas BSG10 showed the lowest (35.1%), followed by BSG40 (42.1%) and BSG20 (45.3%). At −2 and −3 kPa tension, the decrease in VWC continued, with values consistently distributed in proportion to the BSG percentage. At −2 kPa, the values were 0PR (30.1%), BSG10 (26.6%), BSG20 (36.2%), and BSG40 (35.5%). At −3 kPa, the values were 0PR (26.4%), BSG10 (23.9%), BSG20 (33.1%), and BSG40 (32.4%). At a tension of −5 kPa, the rate of water loss slowed down, with values of 0PR (23.5%), BSG10 (21.1%), BSG20 (29.0%), and BSG40 (30.4%). Finally, at the highest tension of −10 kPa, the differences between the substrates further narrowed, with 0PR (22.1%) maintaining the highest value and BSG10 (19.9%) the lowest. BSG20 (27.9%) and BSG40 (29.2%) exhibited significantly higher water retention.

### 2.3. Chemical Characteristics of Substrates

Table 2 presents the ANOVA results for the effects of peat replacement (PR) with wood fiber (WF), coffee silverskin (CS), and brewer’s spent grain (BSG) on chemical properties, pH and EC (electrical conductivity) of the substrates assessed before cultivation.

**Figure 9:**
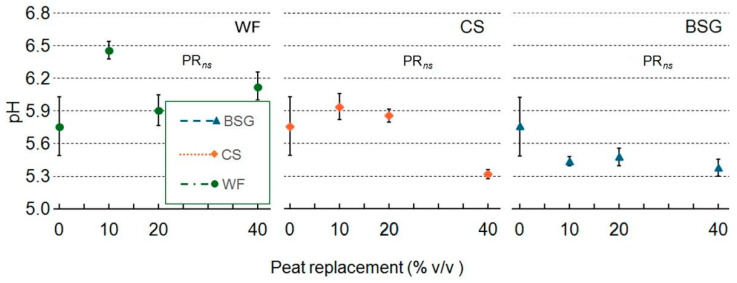
Effect of peat replacement level (PR, 0–40% *v*/*v*) with three by-product materials—wood fiber (WF), coffee silverskin (CS), or brewer’s spent grain (BSG)—on pH of the final substrate mixture. Data points represent means ± standard error (n = 3). Statistical analysis performed through ANOVA: ns, not significant.

Moving on to examine Table 2, no significant effect on pH was observed following the PR with any of the substrate types. The pH values remained relatively stable across all PR levels for WF, CS, and BSG, with statistical analysis confirming no significant trends (PR*_ns_*) in all cases (Figure 9).

**Figure 10:**
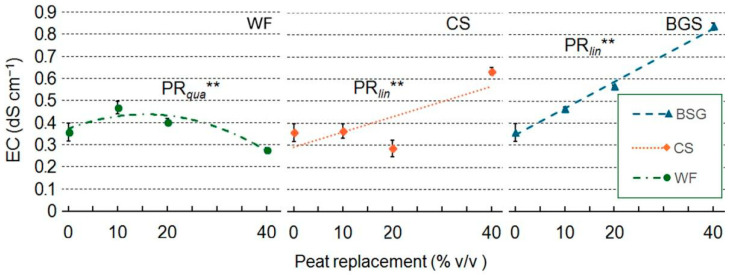
Effect of peat replacement level (PR, 0–40% *v*/*v*) with three by-product materials—wood fiber (WF), coffee silverskin (CS), or brewer’s spent grain (BSG)—on the electrical conductivity (EC) of the final substrate mixture. Data points represent means ± standard error (n = 3). Statistical analysis performed through ANOVA: **, significant at *p* ≤ 0.01. Orthogonal polynomial contrasts were employed to evaluate linear (*lin*) and quadratic (*qua*) trends.

When WF was used as a substrate (Figure 10), the trend was asymptotic; its interpretation remains uncertain, probably due to the limited dataset available. For CS, EC remains relatively stable at lower PRs; the 20% PR appears to be an outlier. EC begins to increase more noticeably beyond 30%, following a general linear pattern. In contrast, BSG shows a strong linear increase in EC with increasing peat replacement, reaching the highest EC value at PR40 (Figure 10).

### 2.4. Sage Growth

Table 3 (cv. *Victoria*) and Table 4 (cv. *Amistad*) report the ANOVA results for the effect of Substrate Type (ST) and Peat Replacement (PR) on sage growth parameters at the end of the vegetative phase (14 January 2024).

**Figure 11:**
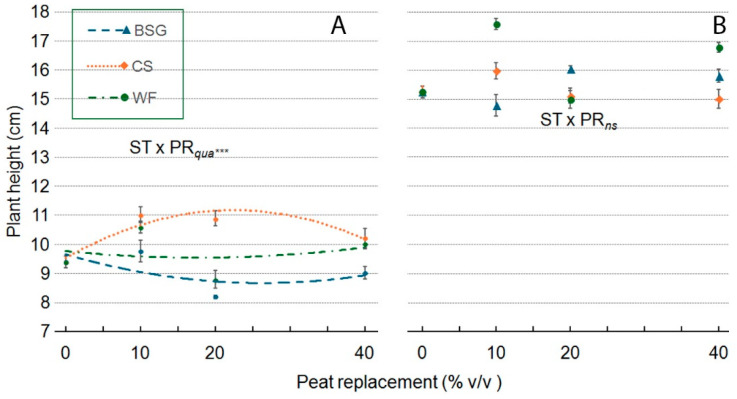
Effect of peat replacement level (PR, 0–40% *v*/*v*) with three organic materials (ST)—brewer’s spent grain (BSG), coffee silverskin (CS), or wood fiber (WF)—on plant height (cm) in Victoria (**A**) and Amistad (**B**) genotypes at the end of the vegetative phase (14 January 2024). Data points represent means ± standard error (n = 3). Statistical analysis performed through ANOVA: ns, and ***, not significant or significant at *p* ≤ 0.001, respectively. Orthogonal polynomial contrasts were employed to evaluate quadratic (*qua*) trend.

In ‘Victoria’ (Figure 11A), plant height remained relatively stable across all PR levels when WF was used as the substrate. With the BSG, the plant height showed a quadratic response with a slightly concave pattern. The CS substrate demonstrated similar behavior, but the asymptotic and convex trend, reaching its highest height value at 20–25% PR. In ‘Amistad’, the ST × PR*qua* interaction was not significant (Figure 11B).

**Figure 12:**
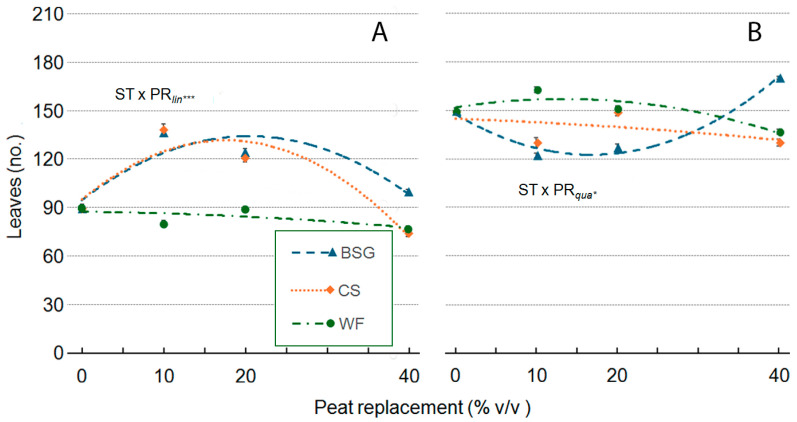
Effect of peat replacement level (PR, 0–40% *v*/*v*) with three alternative organic matrices (ST)—brewer’s spent grain (BSG), coffee silverskin (CS), or wood fiber (WF)—on leaves per plant (no.: number) in ‘Victoria’ (**A**) and ‘Amistad’ (**B**) genotypes at the end of the vegetative phase (14 January 2024). Data points represent means ± standard error (n = 3). Statistical analysis performed through ANOVA: * and ***, significant at *p* ≤ 0.05, or *p* ≤ 0.001, respectively. Orthogonal polynomial contrasts were employed to evaluate linear (*lin*) and quadratic (*qua*) trends.

In ‘Victoria’, PR generally reduced leaf number per plant, although the effect varied by substrate type. With WF, leaf number decreased linearly as the PR percentage increased. In contrast, the CS substrate initially showed a slight increase in leaf number at PR10, followed by a clear linear decline at higher PR levels. The BSG substrate had the least impact, with leaf numbers remaining relatively stable across all PR levels (Figure 12A). Similarly to ‘Victoria’, increasing PR in ‘Amistad’ generally reduced leaf number per plant, with substrate-specific asymptotic trends. (Figure 12B). In ‘Victoria’ (Figure 12A), the number of leaves per plant was negatively affected by wood fiber (WF) at all substitution levels, from −11% at PR10 to −14% at PR40, whereas coffee silverskin (CS) strongly increased leaf number at PR10 and PR20 (+54 and +34%), but declined at PR40 (−17%). Brewer’s spent grain (BSG) enhanced leaf formation at PR10 and PR20 (+52% and +38%), with only a minor increase at PR40 (+11%). In ‘Amistad’ (Figure 12B), the number of leaves per plant, when WF was PR, slightly increased under PR10 (+9%), and decreased at PR40 (−8%). CS reduced leaf number both at PR10 and PR40 (−13%). BSG decreased leaf numbers at PR10 and PR20 (−18 and −15%) but markedly increased at PR40 (+14%).

**Figure 13:**
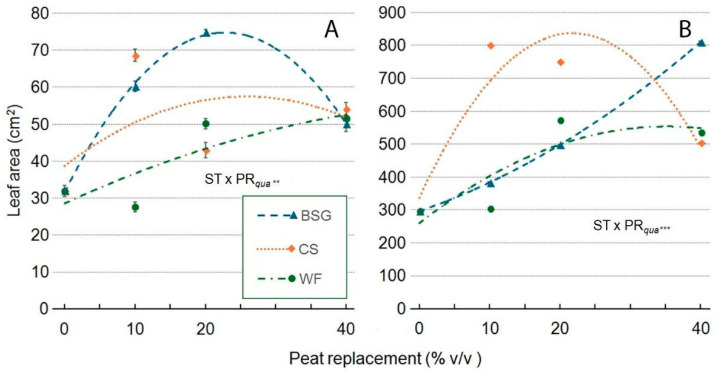
Effect of peat replacement level (PR, 0–40% *v*/*v*) with three alternative organic matrices (ST)—brewer’s spent grain (BSG), coffee silverskin (CS), or wood fiber (WF)—on leaf area (cm^2/plant^) in ‘Victoria’ (**A**) and ‘Amistad’ (**B**) genotypes at the end of the vegetative phase (14 January 2024). Data points represent means ± standard error (n = 3). Statistical analysis performed through ANOVA: **, and ***, significant at *p* ≤ 0.01, or *p* ≤ 0.001, respectively. Orthogonal polynomial contrasts were employed to evaluate quadratic (*qua*) trend.

Regarding leaf area per plant, as the primary metric of leaf development, when WF was used as the substrate, it consistently increased with higher PR levels. However, this effect was less pronounced than that of the other treatments. The response followed a near-linear to slightly quadratic positive trend in both genotypes, indicating that WF moderately benefited leaf area development. With a BSG substrate, leaf area exhibited a quadratic response, peaking at approximately 20–30% PR in ‘Victoria’ and up to PR40 in ‘Amistad.’ The trend was convex for ‘Victoria’ and slightly concave for ‘Amistad.’ CS exhibited a positive trend similar to that of BSG but required lower PR levels to achieve comparable effects. In ‘Victoria’, leaf area reached its maximum at approximately 20% before declining, whereas in ‘Amistad’, it peaked at 30%. (Figure 13A,B). In ‘Victoria’ (Figure 13A), WF reduced plant leaf area to PR10 (−13%) but markedly increased it at 20 and 40 PR levels (+50% and +61%, respectively). CS, on the one hand, strongly promoted it at PR10 (+115%), while on the other hand, it was moderately promoted at PR20 (+34%) and PR40 (+69%). BSG caused substantial increases at PR10 (+88%) and PR20 (+134%), followed by a less pronounced improvement at PR40 (+57%). Conversely, ‘Amistad’ (Figure 13B) responded positively to all substrate types. WF showed strong increases at both PR20 (+93%) and PR40 (+81%), while CS induced the most significant gains at both PR10 (+170%) and PR20 (+153%), and a moderate increase at PR40 (+69%). BSG promoted progressive improvements up to PR40 (+173%).

**Figure 14:**
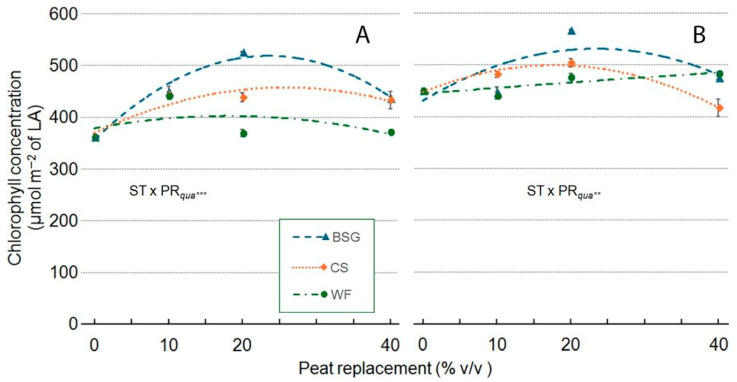
Effect of peat replacement level (PR, 0–40% *v*/*v*) with three alternative organic matrices (ST) —brewer’s spent grain (BSG), coffee silverskin (CS), or wood fiber (WF)—on Chlorophyll Concentration (ChlC) in ‘Victoria’ (**A**) and ‘Amistad’ (**B**) genotypes at the end of the vegetative phase (14 January 2024). LA: leaf area. Data points represent means ± standard error (n = 3). Statistical analysis performed through ANOVA: ** and ***, significant at *p* ≤ 0.01, or *p* ≤ 0.001, respectively. Orthogonal polynomial contrasts were employed to evaluate quadratic (*qua*) trend.

With WF as substrate, ‘Victoria’ Chlorophyll Concentration (ChlC), exhibited a quadratic response, with values peaking at 20% PR before declining at higher replacement levels (Figure 14A). In ‘Amistad’, ChlC showed a slight near-linear increase as the WF proportion increased, with no observed peak (Figure 14B). With CS as substrate, both genotypes reached maximum ChlC values at PR20. At PR40, the ChlC of ‘Victoria’ remained slightly above the control (0PR), while in ‘Amistad’, it dropped below control levels. With BSG, both genotypes displayed an asymptotic response, with ChlC values peaking at PR20, followed by a sharp decline at higher doses (Figure 14A,B). In ‘Victoria’ (Figure 14A), WF produced consistent increases across all levels: +10% (PR10), +20% (PR20), and +9% (PR40). CS stimulated growth moderately, from +4% (PR10) to +16% (PR40). BSG, however, showed a slight effect at PR10 (+2%), a more substantial increase at PR20 (+16%), and a truly impressive increase at PR40 (+25%). ‘Amistad’ (Figure 14B), instead, displayed more variable responses. WF reduced ChlC at PR10 and PR20 (−2% and −7%, respectively) but slightly promoted it at PR40 (+1%). CS markedly enhanced ChlC at PR10 (+13%), while producing small reductions at PR20 and PR40 (−2% and −6%, respectively). BSG decreased ChlC at PR10 (6%) but strongly stimulated it at higher levels (+29% at PR20 and +40% at PR40).

**Figure 15:**
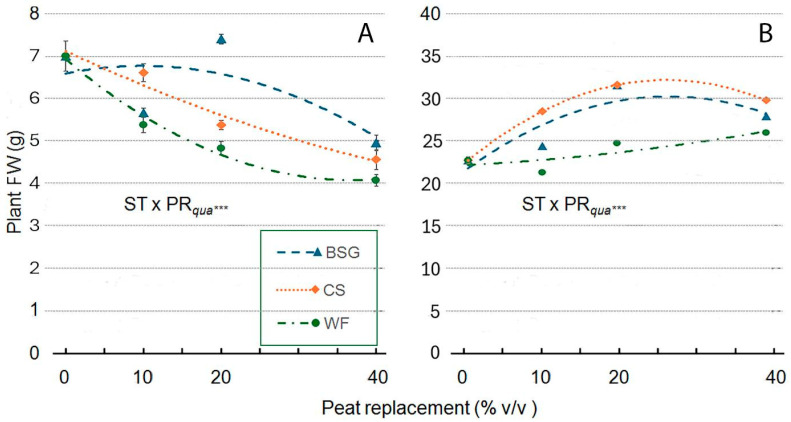
Effect of peat replacement level (PR, 0–40% *v*/*v*) with three alternative organic matrices (ST) —brewer’s spent grain (BSG), coffee silverskin (CS), and or wood fiber (WF)—on Plant Fresh Weight (FW) in ‘Victoria’ (**A**) and ‘Amistad’ (**B**) genotypes at the end of the vegetative phase (14 January 2024). Data points represent means ± standard error (n = 3). Statistical analysis performed through ANOVA: ***, significant at *p* ≤ 0.001. Orthogonal polynomial contrasts were employed to evaluate the quadratic (*qua*) trend.

Plant fresh weight (Plant FW) is a key trait in ornamentals as it integrates growth and resource-use efficiency. Higher values reflect greater biomass, visual quality, and vigor, directly enhancing marketability and consumer appeal. In both genotypes, plant fresh weight was significantly influenced by substrate type and PR level, with notable differences between ‘Victoria’ and ‘Amistad’ (Figure 15A,B). In the ‘Victoria’ genotype (Figure 15A), increasing the PR level with the three organic by-products generally decreased plant fresh weight; the response was non-linear for all substrates, with trends suggesting an asymptotic approach. In the ‘Amistad’ genotype (Figure 15B), the trend was the opposite. With WF as substrate, fresh weight peaked at 40% PR. CS and BSG, on the other hand, showed a different trend from WF as described by the curves: the fresh weight value increased steadily reaching a maximum at 25% PR and then decreased slightly. In ‘Victoria’ (Figure 15A), the replacement of peat with wood fiber (WF) caused a progressive plant fresh weight decline already at PR10 (−23%), with further reduction at PR20 (−31%) and the most severe decrease at PR40 (−42%). A similar trend was observed when coffee silverskin (CS) replaced peat, resulting in a reduction from 6% (PR10) to 35% (PR40). Brewer’s spent grain (BGS) showed a more variable effect, with a reduction at PR10 (−19%), a slight increase at PR20 (+6%), and a strong decrease at PR40 (−29%). Conversely, ‘Amistad’ (Figure 15B) demonstrated a more favorable and consistent response to substrate replacement: WF increased both to PR20 (+9%) and PR40 (+14%); CS promoted significant stimulation at PR10 (+25%), maximum at PR20 (+38%), and still positive effects at PR40 (+30%). When BGS replaced peat, it also enhanced plant fresh weight, with a modest increase at PR10 (+7%), a maximum at PR20 (+38%), and sustained improvement at PR40 (+22%).

**Figure 16:**
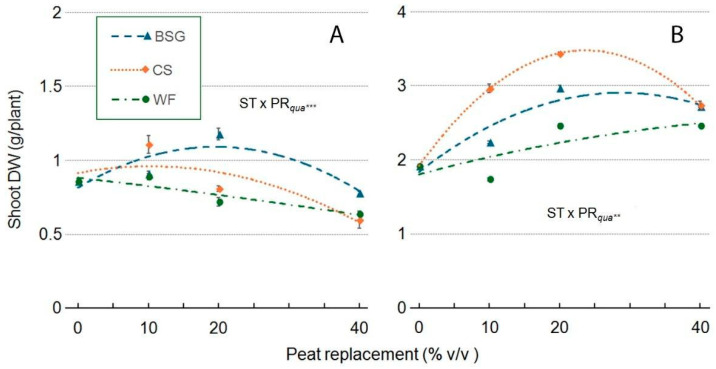
Effect of peat replacement level (PR, 0–40% *v*/*v*) with three alternative organic matrices (ST) —brewer’s spent grain (BSG), coffee silverskin (CS), or wood fiber (WF)—on shoot dry weight (g) in ‘Victoria’ (**A**) and ‘Amistad’ (**B**) genotypes at the end of the vegetative phase (14 January 2024). Data points represent means ± standard error (n = 3). Statistical analysis performed through ANOVA: ** and ***, significant at *p* ≤ 0.01, or *p* ≤ 0.001, respectively. Orthogonal polynomial contrasts were employed to evaluate quadratic (*qua*) trend.

With WF substrate, shoot dry weight (shoot DW) exhibited a quadratic response: in ‘Victoria’, it consistently decreased with the highest PR level (40%), whereas in ‘Amistad’, the trend was the opposite (Figure 16A,B). The CS substrate, initially, in ‘Victoria’, showed a slight increase in shoot DW at 10% PR, followed by a clear quadratic decline at higher PR levels (Figure 16A). In ‘Amistad’, shoot DW peaked at approximately PR20 (Figure 15B). When BSG was used as the substrate, shoot DW exhibited a different quadratic response: in both genotypes, 20PR showed the highest value; over PR20, in ‘Victoria’, it decreased to a value below the control (0PR); in ‘Amistad’, shoot DW decreased slightly with PR40 (Figure 16A,B). In the ‘Victoria’ genotype (Figure 16A), WF resulted in a slight increase in shoot dry weight at PR10 (+3%), but it decreased dry weight at both PR20 (−16%) and PR40 (−25%). CS moderately stimulated dry weight at PR10 (+28%) but caused reductions at PR20 (−6%) and PR40 (−30%). BSG had a mild positive effect on above-ground dry weight at PR10 (+5%), a substantial increase at PR20 (+37%), and a slight reduction at PR40 (−9%). In contrast, the ‘Amistad’ genotype (Figure 16B) showed overall stimulation across the different substrates. WF increased biomass at PR20 and PR40 (+28% each), although it led to a slight decrease at PR10 (−9%). CS significantly promoted growth at PR10 (+55%) and PR20 (+79%), with a moderate increase at PR40 (+43%). BSG demonstrated gradual improvements in biomass, with increases of +17% at PR10, +55% at PR20, and +42% at PR40.

**Figure 17:**
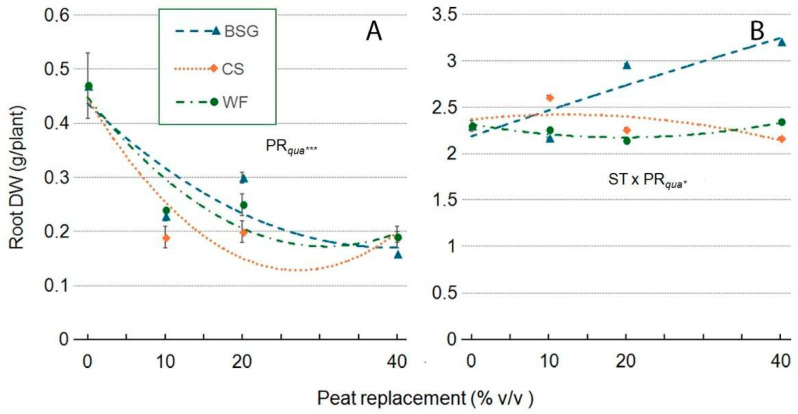
Effect of peat replacement level (PR, 0–40% *v*/*v*) with three alternative organic matrices (ST)—brewer’s spent grain (BSG), coffee silverskin (CS), or wood fiber (WF)—on root dry weight (g) in ‘Victoria’ (**A**) and ‘Amistad’ (**B**) genotypes at the end of the vegetative phase (14 January 2024). Data points represent means ± standard error (n = 3). Statistical analysis performed through ANOVA: * and ***,significant at *p* ≤ 0.05, or *p* ≤ 0.001, respectively. Orthogonal polynomial contrasts were employed to evaluate quadratic (*qua*) trend.

In both genotypes, root dry weight (Root DW) was significantly influenced by PR and substrate type, with notable differences between ‘Victoria’ and ‘Amistad’ (Figure 17A,B). In ‘Victoria’, root DW showed a concave trend in all substrates as PR levels increased (Figure 17A). In ‘Amistad’, the response was substrate-dependent and varied significantly. With BSG, root DW showed a consistently positive trend, increasing with higher PR percentages and peaking at PR40. In contrast, root dry weight remained relatively stable across PR levels for WF and CS, with only minor fluctuations in the latter.

In ‘Victoria’ (Figure 17A), root dry weight was consistently reduced under all treatments, with WF causing declines of −50% (PR10), −48% (PR20), and −61% (PR40). Similarly, CS induced strong reductions (−60%, −58%, −59%), while BSG led to decreases of −51% (PR10), −36% (PR20), and −67% (PR40), highlighting overall limitations in root development regardless of substrate. On the other hand, ‘Amistad’ (Figure 17B) showed a more resilient response: WF slightly decreased at PR10 (−2%) and PR20 (−7%) but slightly increased at PR40 (+1%). CS promoted a moderate increase at PR10 (+13%), while PR20 (−2%) and PR40 (−6%) remained nearly unchanged or experienced slight reductions. BSG, however, clearly enhanced root weight, with improvements at PR20 (+29%) and PR40 (+39%), despite a minor reduction at PR10 (−6%).

**Figure 18:**
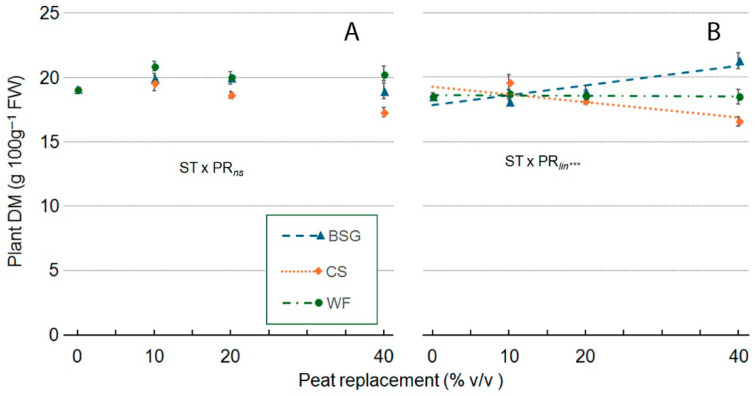
Effect of peat replacement level (PR, 0–40% *v*/*v*) with three alternative organic matrices (ST)—brewer’s spent grain (BSG), coffee silverskin (CS), or wood fiber (WF)—on plant dry matter content (g 100 g^−1^ FW) in ‘Victoria’ (**A**) and ‘Amistad’ (**B**) genotypes at the end of the vegetative phase (14 January 2024). Data points represent means ± standard error (n = 3). Statistical analysis performed through ANOVA: ns, and ***, not significant or significant at *p* ≤ 0.001, respectively. Orthogonal polynomial contrasts were employed to evaluate linear (*lin*) trend.

Regarding plant dry matter (Plant DM) content, the ST × PR interaction was not significant in ’Victoria’ (Figure 18A,B). In ‘Amistad’, the WF substrate had a relatively stable plant DM across all PR levels (Figure 18A). In contrast, the CS substrate initially showed a clear linear decline at a 10% PR level. With BSG substrate as PR, plant DM exhibited a linear response as PR levels increased (Figure 18B).

### 2.5. Leachate pH and EC

Table 5 and Table 6 show the ANOVA results on the effect of Substrate type (ST) and PR on leachate pH and EC values on 15 December 2023, in ’Victoria’ and ‘Amistad’ genotypes.

In the ‘Victoria’ genotype, highly significant effects were observed for both main factors and their interactions, with significance levels (*p* ≤ 0.001) for the pH. Regarding the EC parameter, only ST × PR*qua* was significant at *p* ≤ 0.05, while all the others were significant at *p* ≤ 0.001 (Table 5).

Regarding the ‘Amistad’ genotype, Table 6 shows a highly significant main effect of PR on both pH and EC, while ST significantly influenced pH but not EC. Polynomial contrasts were employed, indicating strong linear and quadratic trends for both response variables. Interaction effects between ST and PR, including their respective linear and quadratic components, were not statistically significant, suggesting that the influence of peat replacement was consistent across substrate types.

**Figure 19:**
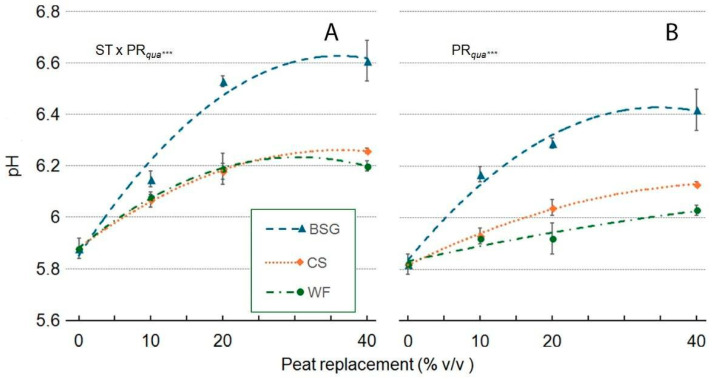
Effect of peat replacement level (PR, 0–40% *v*/*v*) with three alternative organic matrices (ST)—brewer’s spent grain (BSG), coffee silverskin (CS), or wood fiber (WF)—on leachate pH values in ‘Victoria’ (**A**) and ‘Amistad’ (**B**) genotypes. (Data collected on 15 December 2023). Data points represent means ± standard error (n = 3). Statistical analysis performed through ANOVA: ***, significant at *p* ≤ 0.001. Orthogonal polynomial contrasts were employed to evaluate quadratic (*qua*) trend.

In the ‘Victoria’ genotype (Figure 19A), increasing the PR level with the three organic by-products generally increased the leachate pH. The response was non-linear for all substrates, with trends suggesting an approach to an asymptotic maximum. The highest pH value was observed with BSG at PR40. For WF, the pH increased sharply to a maximum at PR25-30 and then appeared to plateau. For CS, the pH increased steadily, reaching its highest value at PR35-40. Similarly, in the ‘Amistad’ genotype (Figure 19B), increasing PR doses raised the leachate pH, with distinct patterns for each substrate. When WF was used, the pH increased in a strong linear pattern with increasing PR percentage. In contrast, the responses for CS and BSG were non-linear and best described by curves. The pH in the CS treatment rose steadily to a maximum at PR40, while the pH in the BSG treatment increased rapidly, peaking at PR30-35 before showing signs of plateauing.

**Figure 20:**
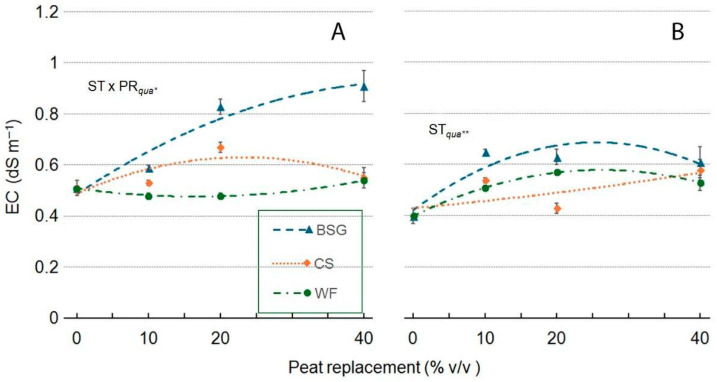
Effect of peat replacement level (PR, 0–40% *v*/*v*) with three alternative organic matrices (ST)—brewer’s spent grain (BSG), coffee silverskin (CS), or wood fiber (WF)—on leachate EC values in ‘Victoria’ (**A**) and ‘Amistad’ (**B**) genotypes. (Data collected on 15 December 2023). Data points represent means ± standard error (n = 3). Statistical analysis performed through ANOVA: * and **, significant at *p* ≤ 0.05 or *p* ≤ 0.01, respectively. Orthogonal polynomial contrasts were employed to evaluate quadratic (*qua*) trend.

In the ‘Victoria’ genotype, the leachate EC value was influenced by ST, and the response was much more varied (Figure 20A). When BSG partially replaced peat, the EC of the leachate showed a asymptotic maximum trend. In contrast, the EC values showed a quadratic response when WF and CS were used as substrate. The quadratic trend was convex in CS, peaking at around 20%, and slightly concave in WF. In the ‘Amistad’ genotype, in general, PR increased the EC values of the leachate: in CS as PR, it increased steadily with higher PR percentages until reaching its maximum at CS40. When WF and BSG replaced peat, the response was quadratic with a convex trend for both, peaking at PR25 in both WF and BSG treatments (Figure 20B).

### 2.6. Principal Component Analysis (PCA) in Growth Parameters

**Figure 21:**
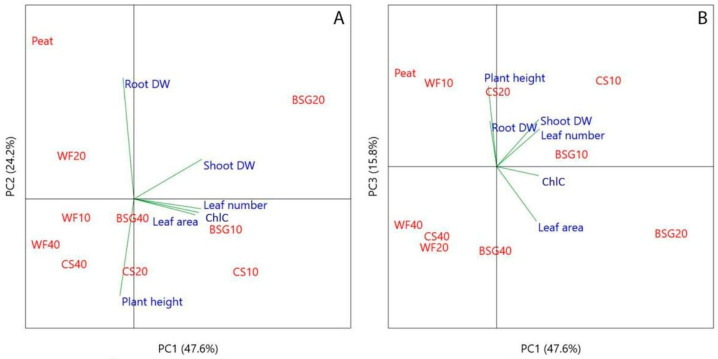
Principal component analysis (PCA) biplot of the PC1 vs. PC2 (**A**) and PC1 vs. PC3 (**B**) principal components, illustrating the relationships between growth parameters and peat replacement treatments in the ‘Victoria’ genotype. The analysis includes shoot dry weight (Shoot DW), root dry weight (Root DW), leaf area per plant, leaf number per plant, plant height, and chlorophyll concentration (ChlC). Substrate treatments are denoted as follows: Peat (0% replacement, control), wood fiber (WF10, WF20, WF40 for 10, 20, and 40% peat replacement, respectively), coffee silverskin (CS10, CS20, CS40 peat replacement), and brewer’s spent grain (BSG10, BSG20, BSG40 peat replacement).

**Figure 22:**
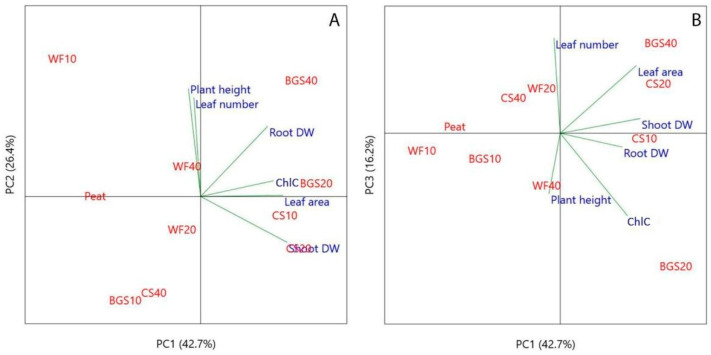
Principal component analysis (PCA) biplot of the PC1 vs. PC2 (**A**) and PC1 vs. PC3 (**B**) principal components, illustrating the relationships between growth parameters and peat replacement treatments in the ‘Amistad’ genotype. The analysis includes shoot dry weight (Shoot DW), root dry weight (Root DW), leaf area per plant, leaf number per plant, plant height, and chlorophyll concentration (ChlC). Substrate treatments are denoted as follows: Peat (0% replacement, control), wood fiber (WF10, WF20, WF40 for 10, 20, and 40% replacement, respectively), coffee silverskin (CS10, CS20, CS40), and brewer’s spent grain (BSG10, BSG20, BSG40).

Principal component analysis (PCA) provided an overview of the bio-morphological traits of ‘Victoria’ (Figure 21A,B) and ‘Amistad’ (Figure 22A,B) sage genotypes.

The PCA performed on the normalized ‘Victoria’ data revealed three principal components (PCs) with eigenvalues >1 that explained approximately 97.6% of the total variance in the dataset, attributing 81.8% to PC1 and PC2. In the depiction of components 1 and 2 (Figure 21A), most variables were positively correlated with PC1, specifically leaf number (r = 0.51), leaf area (r = 0.47), chlorophyll concentration (r = 0.50), and shoot dry weight (r = 0.50). Root dry weight (Root DW) and plant height were negatively correlated with PC1. PC2 positively correlated with shoot dry weight (Shoot DW) and particularly root DW (0.75). A negative correlation with PC2 was detected mainly for plant height (−0.60). The PC3 (Figure 21B) showed a positive correlation mainly with plant height (0.66) and a negative correlation with leaf area per plant (−0.44).

The PCA performed on the normalized ‘Amistad’ data revealed three PCs with eigenvalues >1 that explained approximately 75.3% of the total variance in the dataset, attributing 69.1% to PC1 and PC2. In the depiction of components 1 and 2 (Figure 22A), leaf area per plant (0.52), ChlC (0.47), shoot DW (0.56) and root DW (0.43) were positively correlated with PC1. PC2 showed positive correlations with plant height (r = 0.64) and leaf number per plant (r = 0.59). The PC3 (Figure 22B) showed a positive correlation mainly with leaf number (0.61) and leaf area (0.43), while exhibiting a negative correlation with leaf ChlC (−0.53) and plant height (−0.39).

**Figure 23:**
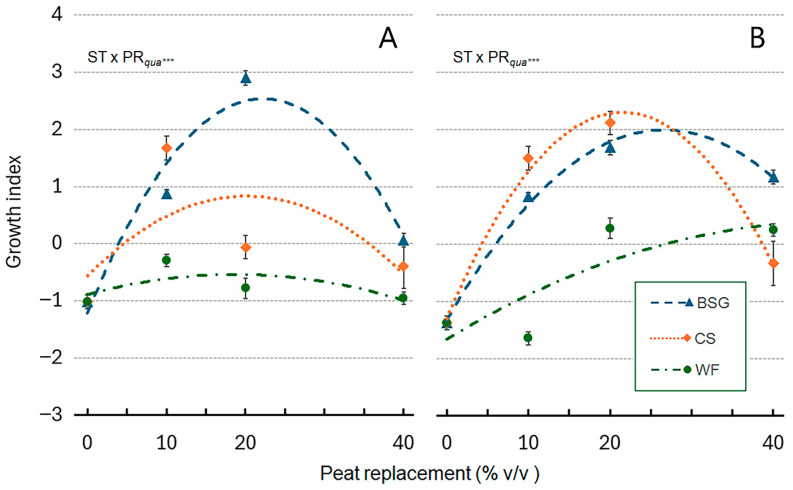
Effect of peat replacement level (PR, 0–40% *v*/*v*) with three alternative organic matrices (ST)—brewer’s spent grain (BSG), coffee silverskin (CS), or wood fiber (WF)—on growth index in ‘Victoria’ (**A**) and ‘Amistad’ (**B**) genotypes. (Data collected on 15 December 2023). Data points represent means ± standard error (n = 3). Statistical analysis performed through ANOVA: ***, significant at *p* ≤ 0.001, respectively. Orthogonal polynomial contrasts were employed to evaluate quadratic (*qua*) trends.

The analysis of the PC1-derived Growth Index (GI) scores revealed for ‘Victoria’, significant differences between treatments. The BSG20 treatment yielded the highest mean GI score, significantly outperforming the peat control (0PR) and all other amendments. Treatments such as CS10 and BSG10 showed moderate GI scores; in contrast, high replacement rates (WF40, CS40, BSG40), 0PR and WF resulted in the lowest GI scores (Figure 23A). For ‘Amistad’, the analysis of the GI scores revealed that the CS20 and BSG20 treatments yielded the highest overall growth, with GI scores significantly greater than those of the 0PR. CS10 and BSG40 also showed strong performance, with high GI scores. WF10 had GI scores similar to PR0, whereas the GI of WF20 and WF40 were slightly higher (Figure 23B).

## 3. Discussion

### 3.1. Physical and Hydrological Characterization

The hydraulic properties of growing media provide precise information about their ability to provide good plant growth conditions [61,62].

#### 3.1.1. Wood Fiber as Peat Replacement

Wood’s by-products, in the form of processed wood fractions, have been tested and applied, for some time now, as promising growing media ingredients and their market has been expanded worldwide [63] due to renewability [7], reduced carbon footprint compared to peat or other materials [64]. Furthermore, it also has a low production cost [65], especially for conifer by-products, which show a lower phytotoxic molecule content compared to hardwood species [66]. In our results, water volume (WV) at –1 kPa, reflecting the moisture retained against minimal suction and hence indicative of easily plant-available water, exhibited a quadratic response to increasing wood fiber (WF) rates (Figure 1). This pattern is consistent with quicker drainage and higher air content (AC) [67,68,69,70] compared to peat. This behavior would appear to result from the coarser and more heterogeneous particle size distribution of WF, which accelerates drainage and reduces water retention at low tension levels [71,72]. Complementary research [54] regarding a 60:40 peat: WF blend revealed that WF exhibits hydrophilic behavior across a range of moisture contents, maintaining contact angles below 90°, unlike peat, improving rewetting dynamics under dry conditions. Substrate moisture (SM), measured at –1 kPa, declined linearly with increasing WF content (Figure 2). This contrasts with peat, which possesses superior water-holding capacity due to its high surface area and microporous structure [13]. The reduced moisture in WF-based media reflects its lower capacity for capillary water retention and is consistent with findings by Gruda [7], who noted a faster drying rate in WF-amended substrates. Both easily available water (Figure 3) and available water (Figure 4) demonstrated a linear decline with increasing levels of wood fiber. While peat typically holds large volumes of water in the range of −1 to −10 kPa—considered optimal for plant uptake—WF was less effective in maintaining this fraction, resulting in a lower easily available water value. Fields et al. [73] found that blends containing pine wood chips or shredded pine wood exhibited steeper moisture retention curves, indicating a sharper decline in volumetric water content (WVC) as suction increased. Pure wood substrates displayed higher drainage rates and reduced retention in the −1 to −30 kPa range. These findings reinforce that wood components have lower water-holding capacity (WHC) and faster drying tendencies than peat blends. Durand et al. [74] and Atzori et al. [23] explain this behavior: WF substitution often leads to reduced water availability due to diminished micropore volume and lower capillary retention. According to Michiels et al. [75], an ideal substrate has less easily available water of <40 vol.%. The values of all WF-based substrates for nursery production were within the range of an ideal substrate, suggesting that they can be effectively integrated without severely compromising substrate functionality. Unlike other peat substitutes, such as coffee silverskin (CS) or brewer’s spent grain (BSG), WF did not significantly affect buffering capacity (BC) (Figure 5). The inherent lignocellulosic structure and minimal nutrient content of WF may contribute to its low interaction with substrate pH and nutrient fluxes, corroborating Zaccheo et al. [24]. A linear increase in dry bulk density (BD, Figure 6), accompanied by a corresponding linear decrease in total porosity, a key determinant of air–water balance in container media (Figure 7), was observed with increasing levels of WF replacement. While peat possesses a naturally spongy and lightweight structure [76], WF—although also lightweight—contains more rigid particles that may compact under load, thereby reducing pore volume [5]. Despite these changes, dry BD values remained within the acceptable horticultural range (<0.4 g·cm^−3^), indicating no significant risk of substrate compaction. The water retention profiles (Figure 8A) further illustrated that increasing WF content led to a steeper decline in volumetric water content (WVC) at increasing tensions. While the control 0PR maintained higher WVC across all tensions, the WF-based substrate (especially at 40% of peat replacement) exhibited more rapid water loss between −1 and −5 kPa tension. This underscores the limited capillary continuity and weaker water-holding ability of WF, consistent with findings from Gruda and Schnitzler [68], Wallach [2] and Muhammed et al. [36]. Eveleens et al. [77], too, when they added 40 and 50% *v*/*v* WF to peat, showed that these substrates reduce WVC by 16% *v*/*v* and increase AC by the same amount in the range of −6 to −100 cm pressure head. Our results regarding the water retention curves (Figure 8A) reflect the lower capillary water retention capacity in substrates with a higher WF replacement level. WF’s coarse texture and lower micropore volume limit its ability to retain water against even modest tension, consistent with prior findings [54,73,77]. The smaller differential at high tension suggests that WF substitution primarily affects water availability within the plant-easily available range (–1 to –5 kPa), while the residual water at high tension remains similar across media types. Our findings are in line with previous studies showing that high proportions of WF (>30%) significantly reduce easily available water (EAW) and total available water (TAW) due to increased macroporosity and limited fine-pore storage [7,70]. Notably, Michel et al. [54] reported that while WF improved rewetting dynamics, it did so at the cost of moisture retention in the root-active zone. Regarding chemical characteristics, pH and electrical conductivity (EC) are two important factors affecting the growing medium due to the direct effect on nutrient availability and status in the growing medium [78]. In our research, the pH value in the WF-based substrate was n.s. (Figure 12); this agrees with Woznicki et al. [79], who report that substrates with over 50% (*v*/*v*) WF showed a rapid pH increase.

#### 3.1.2. Coffee Silverskin as Peat Replacement

In our study, the proportional peat replacement (PR) with CS underscores a potential decline in capillary WHC due to the inclusion of CS. References reported that CS has a high content of recalcitrant lignocellulosic compounds that contribute to bulk and physical structure in substrates [80,81]. Other authors [82,83] have linked the WHC of CS alone to the soluble fiber content. The physical behavior of substrates incorporating CS is strongly influenced by its unique particle morphology. As reported by Miler et al. [26], the flat and delicate flake-like structure of CS, in contrast to more fibrous peat or WF, contributes to increased compaction within the substrate matrix. This morphological trait hinders the optimization of the water–air balance, a key determinant of substrate performance. Although CS exhibits low BD, its limited structural resilience may reduce pore continuity and restrict gaseous exchange in the root zone. This is consistent with previous findings that link excessive substrate compaction and insufficient macroporosity to reduced oxygen availability and impaired root respiration [76,84]. Similar trends have been observed with other finely textured or plate-like organic materials, which often exhibit poor structural stability under cultivation conditions [85,86]. Consequently, substrates with high CS content may limit root zone aeration, potentially compromising plant growth and quality.

#### 3.1.3. Brewer’s Spent Grain as Peat Replacement

To date, no studies have comprehensively characterized the physical-hydrological properties of growing media formulated with BSG, highlighting a significant gap in the current literature. In contrast, existing research on raw material itself provides some foundational insights. For instance, Naibaho et al. [87] reported bulk density values for BSG ranging between 0.129 and 0.159 g cm^−3^, while Castro and Colpini [88] documented a consistently high moisture content, typically between 78% and 84%. This substantial water content is primarily attributed to the structural and compositional characteristics of BSG. The fibrous matrix is notably rich in cellulose, hemicellulose, and lignin—components that enhance water retention through both hydrogen bonding and capillary entrapment mechanisms. Among these, hemicellulose is particularly hydrophilic, enabling substantial water adsorption within the porous network of the BSG [89]. Furthermore, the mashing process in brewing, which partially disrupts plant cell walls, increases the porosity and specific surface area of the biomass, thereby improving its WHC [90]. In addition to structural polysaccharides, residual proteins and gelatinized starch fractions present in BSG may contribute to moisture stabilization by forming hydrated matrices that interact with water molecules [91].

### 3.2. Chemical Characteristics of Growing Media 

The chemical stability and ionic activity of growing media are pivotal factors influencing plant performance in containerized horticultural systems. Among the key physicochemical parameters, pH and EC serve as critical indicators of substrate suitability, directly affecting nutrient solubility, microbial dynamics, and root development. Across all tested PR levels (10–40%), no statistically significant variation in pH was observed for WF, CS, or BSG treatments (Figure 9). This pH stability underscores the dominant BC of peat, which remains effective even when partially substituted with organic residues [92]. The lignocellulosic nature of WF and CS contributes to their low acid dissociation potential, thereby exerting minimal influence on substrate pH. Although BSG is rich in proteins and amino acids, its limited mineralization before cultivation is likely to delay any acidifying effects, further supporting pH consistency across treatments. In contrast, EC exhibited substrate-specific and dose-dependent variability (Figure 10), reflecting differences in soluble salt concentrations and rates of organic matter decomposition. Notably, BSG treatments demonstrated a strong linear increase in EC with rising replacement levels, reaching the highest values at 40%. This trend is attributed to the elevated concentrations of organic nitrogen, phosphorus, and potassium in BSG, which undergo rapid mineralization and contribute to increased ionic activity in the substrate [93].

### 3.3. Principal Component Analysis (PCA) in Growth Parameters

The principal component analysis (PCA) for ‘Victoria’ sage highlighted that the first two PCA components (Figure 21A) enable the distinction between mixtures with PR10 of both BSG and CS, and those with PR20 of BSG. The latter, located in the first quadrant (upper right), clustered according to shoot DW. While for BSG10 and CS10 mixtures (lower-right quadrant) clustering was driven by leaves leaf number and area, and chlorophyll concentration (ChlC). The PC3 also supported the best performance of BSG20, primarily in terms of leaf area (Figure 21B). These findings could be related to the nutrient availability of BSG, which is rich in organic matter, proteins, nitrogen, phosphorus, and micronutrients. At PR20, BSG may provide an optimal nutrient balance that enhances shoot development without causing substrate compaction or nutrient imbalance. Furthermore, moderate inclusion of BSG can improve aeration (Figure 7), enhance water retention, and increase water availability (Figure 8), thereby supporting shoot expansion and leaf development compared to 0PR. The ‘Victoria’ genotype may be particularly responsive to the nutrient profile or physical properties of BSG at this concentration, suggesting their successful use in the cultivation. Located on the left upper side of the PC1 component, WF20 and mainly peat displayed a high correlation with root DW, while other mixtures (left lower side) mainly improved the plant height. Furthermore, PC3 separated peat (0PR), CS20 and WF10 clustering for root DW and plant height (Figure 21B). WF20 could create a more oxygen-rich rhizosphere, encouraging root elongation and biomass accumulation; on the other hand, BSG may release nutrients more rapidly, favouring shoot elongation and vertical growth rather than root expansion. Peat-based substrates consistently yield higher or comparable root DWs and overall plant growth, especially when compared to high-percentage WF or alternative organic amendments [94,95,96]. In general, our results show that in ‘Victoria’, 0PR clustered distinctly, reflecting optimal growth conditions. When included at moderate levels (10–20 PR), WF maintains structural stability and high macroporosity, supporting strong root aeration and growth [68], alignment with WF10-20 closeness to control in PCA. At higher proportions (WF40 as PR), excessive porosity may reduce water retention and nutrient availability, echoing the PCA displacement toward less favourable axes [85,86]. The flake-like, delicate morphology of CS leads to high compaction and a poor air-filled pore space, despite its low Bulk Density—consistent with PCA clusters for CS20 and CS40, which are located far from the growth vectors [26,80]. High recalcitrant lignocellulose content may immobilize nitrogen and slow decomposition, further contributing to poor plant performance at higher inclusion rates [97]. With moderate hydrophilic fiber content, BSG10-20 PR occupies intermediate positions in PCA space, offering improved moisture retention without severe structural issues [89,90]. However, high BSG PR (BSG40) may lead to compaction, rapid microbial decay, and salinity buildup—explaining its divergence in PCA from positive growth traits [98].

The Growth Index (GI) (Figure 23), which represents a PC1-derived index, provided a powerful, unified metric to rank substrate efficacy. It indicates that a 20% replacement of peat with BSG provided the optimal conditions for holistic plant development in ‘Victoria’ (Figure 23A). The biplot (Figure 21A) illustrates how this treatment is positioned farthest along the positive direction of the PC1 axis.

Moderate GI scores of CS10 and BSG10 were due to clustering in the biplot based on strong performance in specific traits like leaf area and chlorophyll, but not all traits simultaneously. The peat control (0PR), the WF, and high replacement rates, with the lowest GI scores, clustered on the negative side of PC1, aligning with their observed poor performance and displacement from positive growth vectors in the biplot. In general, in ‘Victoria’, the lowest GI for WF substrate across nearly all peat replacement levels indicated that it was the most detrimental alternative for this cultivar.

The first two PCA components (Figure 22A) for ‘Amistad’ genotype allow for the distinction of mixtures PR at 10–20 CS, and 20–40 BSG. The BSG20 and BSG40, located in the first quadrant (upper right), clustered according to root DW and ChlC. The findings related to BSG substrates could be related to nitrogen availability, thus richness in organic nitrogen and amino acids, which can stimulate root development, chlorophyll biosynthesis [99] and photosynthetic capacity [93]. In contrast, for CS10 and CS20 PR (lower-right quadrant), clustering was driven by leaf area and shoot DW. This finding could be related to a rapid nutrient release: Melo et al. [100] confirmed that a high caffeine and polyphenolic content can act as biostimulants, promoting shoot elongation and leaf expansion. Furthermore, at low PR levels, CS improves substrate structure without compromising water retention, supporting vigorous shoot development. Located on the left upper side of Component 1, WF10 showed a high correlation with plant height (0.64) and plant leaf number. Furthermore, PC3 separated WF20 for leaf number and WF40 for plant height. WF40 and CS40, located distantly from the 0PR, showed reduced association with both shoot and root biomass, indicating potential limitations in substrate structure or nutrient availability at higher PR. In ‘Amistad’, the best substrates appear to be CS10 and CS20 because they increase shoot weight and plant leaf area, although BSG20 and BSG40 appear to improve root growth and leaf color. Wood fiber at all three concentrations appears to improve some aspects of the above-ground plant, such as height and plant leaf number. In any case, the mixtures seem to perform better than 0PR.

Analysis of GI scores showed a markedly different response from ‘Victoria’. Multiple amendment treatments outperformed the peat control. The CS20 and BSG20 treatments produced the highest overall growth (Figure 23B). CS10 and BSG40 also showed strong performance, with high GI scores, demonstrating ‘Amistad’s’ greater responsiveness and adaptability to peat replacement. This is visually supported by their position in the positive PC1 space of the biplot (Figure 22A). The 10PR treatment with wood fiber generated GI scores comparable to those of the peat control. However, GI for WF20 and WF40 was slightly better than PR0, consistent with their central positioning in the biplot.

In general, the GI-based approach confirms that moderate inclusion of brewer’s spent grain (BSG20) is a highly effective peat replacement for both genotypes, while coffee silverskin (CS) is particularly effective for the ‘Amistad’ genotype. This analysis underscores that optimal substrate formulation is not only dependent on the amendment type and rate but also critically on the plant genotype.

## 4. Materials and Methods

A greenhouse pot experiment was conducted to evaluate the physical characteristics and agronomic performance of alternative substrates, utilizing organic by-products as a partial replacement for peat. Two sage cultivars, characterized by different growth habits, were used as plant test subjects.

### 4.1. Substrate Treatments

Ten substrates were used as treatment. Each substrate included 15% of the total volume of an inorganic fraction and an organic fraction (85% of the total volume). The ten substrates were created by partially replacing peat in the organic fraction with alternative organic matrices. These matrices, selected from by-products of the agricultural industry, included wood fiber (WF), coffee silverskin (CS), and brewer’s spent grain (BSG). Each matrix was used at four levels of peat replacement (0, 10, 20, and 40% *v*/*v* of the organic fraction), resulting in the treatments summarized in Table 7.

The sources of the organic matrices were: (i) peat (P, Plantaflor^®^, Vechta, Germany); (ii) WF (Sunfiber^®^, Nuova Flesan S.r.l., Milano, Italy), (iii) CS (Brunocaffè roasting company, Bari, Italy) and (iv) BSG (Peroni brewery, Bari, Italy). WF derived mainly from conifers, such as pine and fir, by mechanical defibrillation of virgin wood chips; CS from a blend of *Coffea arabica* L. (20%) and *C. robusta* Pierre ex A. Froehner (syn. *C. canephora* 80%), BSG was obtained from the production of a lager beer brewed with barley malt (70%) and maize (30%) and does not contain spent yeast.

Regarding some physical characteristics, such as particle size or fraction, in WF, there was a blend of 3 (10–16 mm), 25 (4–10 mm), 25 (2–4 mm), 9 (1–2 mm), and 38% (<1 mm). Regarding the average particle size, as diameter, in CS it was 1.5–2.0 mm and in BSG it was 3.5 mm. Sdao et al. [98] analyzed the four organic matrices and provided values for EC, TNK and P content in experiments involving bedding plant species in a potted greenhouse cultivation.

### 4.2. Experimental Setup

The on-field cultivation experiment was carried out from 16 September 2023 to 12 February 2024 (150 days) in an unheated and naturally lit greenhouse, covered with ethyl vinyl acetate (EVA) plastic film, located in a commercial ornamental nursery (Monopoli, Bari, Italy, 40°54′19.1″ N, 17°18′21.4″ E; 66 m a.s.l.). The site has a typical Mediterranean climate (climate type “Csa” of the Köppen Classification) and it is characterized by hot, dry summers and cool, wet winters. During the experimental period, the Monopoli climatic station reported average monthly temperatures ranging from 25 to 26 °C (max) and 18–20 °C (min) in September, declining to 12–13 °C (max) and 7–8 °C (min) by February.

Two sage cultivars (‘Victoria’ and ‘Amistad’) were grown in 1.2 L recycled plastic pots filled with the substrates described above. Both cultivars represent high-market value ornamental crops and were also selected for their widespread ornamental application and proven adaptability to Mediterranean climatic conditions. Those two genotypes were chosen for their contrasting growth habits, with ‘Victoria’ exhibiting more compact shoot and root development and ‘Amistad’ showing a more vigorous and expansive growth, long stems with numerous leaves. Table S1 shows the primary botanical and ornamental characteristics, as well as the agronomic needs for the utilization of green spaces, of the two genotypes.

Forty-day-old young transplants (approx. 4.0 cm height), grown in small plastic pots (4 cm × 4 cm × 4 cm) filled with *Sphagnum* peat and obtained from rooted stem soft cutting, were used as plant starting material.

One plant per pot was transplanted on 16 September 2023, using 480 sage plants per genotype. Sixteen pots were used per experimental unit. Plants were spaced 30 cm × 30 cm apart and were arranged on growing benches in a randomized block design with three replications per treatment. Irrigation was supplied through one pressure-compensated dripper per pot, delivering 2 L h^−1^. Raw water from the experimental farm (electrical conductivity = 0.45–0.65 dS m^−1^; pH= 6.3–6.5) was used. Irrigation frequency and duration were controlled by a timer and adjusted throughout the growing period according to prevailing weather conditions, to replenish moisture losses in the substrates, bringing the pots up to a certain weight. Peat was pre-enriched with 4.5 kg m^−3^ calcium carbonate. For mineral nutrition, each pot received a controlled-release fertilizer (N:P:K = 16:8:12 + 2MgO + micronutrients, 6-month release) at a rate of 3 g L^−1^, following the manufacturer’s recommendations (Compo Expert, Italy).

### 4.3. Physical and Hydrological Substrates Characterization

Before transplanting, the substrates were characterized for their main physical properties. These included WV = Water Volume at −1 kPa; AC = Air Content at −1 kPa; SM = Substrate Moisture at −1 kPa; EAW = Easily Available Water; BC = Water Buffering Capacity; TAW = Total Available Water; TPS = Total Porosity Space; BD = Dry Bulk Density. These parameters were determined by constructing water retention curves using a tension table according to the method described by De Boodt et al. [84], and further applied by Abad et al. [86] and Noguera et al. [37] for the physical evaluation of organic soilless substrates. All analyses were conducted in triplicate under controlled laboratory conditions using stainless cylinders and porous plates.

### 4.4. Chemical Substrates Characterization

In this study, pH and electrical conductivity (EC) determinations of the peat control (0PR) substrate and each mixture obtained by WF, CS and BSG as partial peat replacement (PR) were determined before substrate fertilization. Three replications were used to determine the EC [101] and pH [102] of the growing media, which were analyzed in a water extract (substrate sample: distilled water, 1:5 *v*/*v*). Values of pH and EC were measured using a portable XS PC 7 VIO Professional multi-parameter meter (XS Instruments, Carpi, Italy).

### 4.5. Chemical Leachate Substrate Fraction Characterization

The pH and EC of the leached substrate fraction were monitored. The leachate was collected from the pots using the pour-through method [103] one hour after irrigation. A volume of 100 mL of distilled water was poured onto the surface of each pot by hand, and the extract was allowed to drain for 10 min in saucers placed under each container until 50 mL of leachate was obtained. The pH and EC values were measured using a portable multiparameter meter XS PC 7 VIO Professional (XS Instruments, Carpi, Italy) with three replications.

### 4.6. Plant Growth and Biomass Measurements

At the end of the experiment, once the vegetative phase was over, five pots were randomly selected and collected for each replication and each treatment to determine plant growth parameters via non-destructive and destructive sampling. Plant height (cm) and total leaf number (no.) were determined at first. The height of the plant was determined by direct dimensional measurement using a ruler graduated in cm, from the surface substrate level to the apex of the sages. Leaf area per plant (cm^2^) was measured using a Li-Cor area meter (LI-3000, Licor, Lincoln, Nebraska, USA). Chlorophyll concentration (ChlC) was measured using a Chlorophyll Content Meter (Apogee, North Logan, UT, USA) on five selected plants, with three measurements taken per leaf, on six fully intermediate and expanded, mature leaves. Roots were meticulously washed under running tap water, using a sieve to recover any detached fragments. Plant fresh weight (roots and shoots) was recorded using a three-decimal analytical balance and subsequently oven-dried at 80 °C for 48 h in a ventilated oven until constant weight was achieved. Root and shoot dry weights (g) were recorded too. Finally, plant dry matter content (Plant DM) (g 100 g^−1^ fresh weight), was calculated by measuring the fresh weight of individual plant parts (shoots and roots) at the onset of destructive sampling, alongside their corresponding dry weights, using the following formula:

Dry Matter Content (%) = [Dry Weight (g)/Fresh Weight (g)] × 100

### 4.7. Statistical Analysis

All data were analyzed using ANOVA through the GLM Procedure in SAS software (Version 9.0) [104]. Mean values are presented in graphs together with their corresponding standard errors (SE). Orthogonal polynomial contrasts were employed to evaluate linear, quadratic, and cubic trends in response to peat replacement and its interaction with substrate type (ST × PR). Due to the unequal spacing of PR levels (0, 10, 20, and 40%), the trends were analyzed using the following contrast coefficients: linear (−7, −3, 1, 9), quadratic (7, −4, −8, 5), and cubic (3, −8, 6, −1), calculated using the NumPy library in Python (version 3.12.0). Based on theoretical relevance, only biologically meaningful contrasts (e.g., linear or quadratic trends) were reported, even when higher-order trends (e.g., cubic) were statistically significant.

The physicochemical properties of the final substrate mixtures, measured before sage’s transplant, were analyzed separately for each organic matrix type used (WF, CS, or BSG) as PR. A one-way ANOVA was performed with PR level (0, 10, 20, and 40%) as the treatment (three replicates per treatment). The control (0PR) was consistent across all matrices.

Plant growth parameters were evaluated separately for the two genotypes (‘Victoria’ and ‘Amistad’) using a two-way randomized block design, with substrate type (ST) and PR level as factors (three replications per treatment). The control treatment (0PR) was shared across all substrate types.

Plant growth responses were further subjected to Principal Component Analysis (PCA) using PAST 4.03 software (http://folk.uio.no/ohammer/past accessed on 9 July 2025) [105]. Before PCA, all variables were standardized using the formula [(x − mean)/standard deviation] to ensure comparability across measurements. To synthesize the multivariate growth data into a single, biologically meaningful metric, a Growth Index was calculated for each experimental replicate. This index was constructed based on the first principal component (PC1). To ensure the index represented a conserved axis of growth across both cultivars, we included only traits that demonstrated positive loadings on PC1 in both: namely, leaf area, chlorophyll concentration, and shoot dry weight. The final index was computed as the weighted sum of the standardized values (z-scores) for these three traits, with each trait’s contribution weighted by the magnitude of its PC1 loading. The Growth Index data were further subjected to ANOVA.

## 5. Conclusions

The results on the vegetative growth of ornamental sages have highlighted that differential PR rates are required depending on the specific organic by-product and plant genotype. In ‘Victoria’, GI indicates that a 20% replacement of peat with BSG provided the optimal conditions for holistic plant development; the lowest GI for WF substrate across nearly all peat replacement levels indicated that it was the most detrimental alternative for this cultivar. In ‘Amistad’, the analysis of the GI scores revealed that the CS20 and BSG20 of peat replacement yielded the highest overall growth, with GI scores significantly greater than those of the peat control. CS10 and BSG40 also showed high GI scores. WF10 had GI scores similar to those of the peat control.

In general, the GI-based approach confirms that moderate inclusion of brewer’s spent grain (BSG20) is a highly effective peat replacement for both genotypes. At the same time, CS and BSG were particularly effective for the ‘Amistad’ genotype, which was more flexible in adapting to cultivation using the new proposed by-products than the ‘Victoria’ genotype. This analysis underscores that optimal substrate formulation is not only dependent on the amendment type and rate but also critically on the plant genotype. Future research should prioritize analyzing the influence of the chemical characteristics of these by-products on the mineral content of sage genotypes and evaluate sustainability also from the point of view of eco-efficiency and profitability.

## Figures and Tables

**Table 1:** ANOVA results for the effects of peat replacement (PR) with wood fiber (WF), coffee silverskin (CS), or brewer’s spent grain (BSG) on the physical properties of the substrates assessed before cultivation. Orthogonal polynomial contrasts were employed to evaluate linear (*lin*) and quadratic (*qua*) trends. WV = Water Volume at −1 kPa; AC = Air Content at −1 kPa; SM = Substrate Moisture at −1 kPa; EAW = Easily Available Water; BC = Water Buffering Capacity; TAW = Total Available Water; TPS = Total Porosity Space; BD = Dry Bulk Density.

	WV	AC	SM	EAW	BC	TAW	TPS	BD
Wood fiber								
Peat replacement (PR)	* ^a^	ns	*	*	ns	*	***	***
PR*lin*	**	ns	*	*	ns	*	***	***
PR*qua*	*	ns	ns	ns	ns	*	ns	ns
Coffee silverskin								
PR	*	*	***	**	ns	**	***	**
PR*lin*	ns	ns	***	***	ns	***	ns	ns
PR*qua*	ns	ns	**	ns	ns	*	**	**
Brewer’s spent grain								
PR	*	*	*	**	*	***	***	***
PR*lin*	ns	ns	**	***	*	***	***	***
PR*qua*	ns	ns	ns	***	ns	***	***	***

^a^ ns, *, **, and ***, not significant or significant at *p* ≤ 0.05, *p* ≤ 0.01, or *p* ≤ 0.001, respectively.

**Table 2:** ANOVA results for the effects of peat replacement (PR) with wood fiber (WF), coffee silverskin (CS), or brewer’s spent grain (BSG) on chemical properties, pH and EC (electrical conductivity) of the substrates assessed before cultivation. Orthogonal polynomial contrasts were employed to evaluate linear (*lin*) and quadratic (*qua*) trends.

Treatment	pH	EC
Wood fiber		
PR	ns	***
PR*lin*	ns	**
PR*qua*	ns	**
Coffee silverskin		
PR	ns	**
PR*lin*	ns	**
PR*qua*	ns	ns
Brewer’s spent grain		
PR	ns	***
PR*lin*	ns	***
PR*qua*	ns	ns

ns, **, and ***, not significant or significant at *p* ≤ 0.01, or *p* ≤ 0.001, respectively.

**Table 3:** ANOVA results showing the effect of Substrate Type (ST) and Peat Replacement (PR) on the main growth parameters in ‘Victoria’ genotype at the end of the vegetative phase (January 14, 2024). FW: Fresh Weight, DW: Dry Weight, DM: Dry Matter, no.: number; ChlC: Chlorophyll Concentration; LA: leaf area. Orthogonal polynomial contrasts were employed to evaluate linear (*lin*) and quadratic (*qua*) trends.

Treatment	Plant Height (cm)	Leaves (no./Plant)	Leaf Area (cm^2^/Plan)	ChlC (µmol m^−2^ LA)	Plant FW (g/Plant)	Shoot DW (g/Plant)	Root DW (g/Plant)	Plant DM (g 100 g^−1^ FW)
Substrate Type (ST)	*** ^a^	***	***	***	***	***	ns	***
Peat replacement (PR)	***	***	***	***	***	***	***	**
PR*lin*	ns	***	***	***	***	***	***	ns
PR*qua*	ns	***	***	***	ns	*	***	ns
ST × PR	***	***	***	***	***	***	ns	ns
ST × PR*lin*	ns	***	***	***	*	***	ns	ns
ST × PR*qua*	***	ns	***	***	***	***	ns	ns

^a^ ns, *, **, and ***, not significant or significant at *p* ≤ 0.05, *p* ≤ 0.01, or *p* ≤ 0.001, respectively.

**Table 4:** ANOVA results showing the effect of Substrate Type (ST) and Peat Replacement (PR) on plant growth parameters in ‘Amistad’ genotype at the end of the vegetative phase (14 January 2024). FW: Fresh Weight, DW: Dry Weight, DM: Dry Matter, no.: number; ChlC: Chlorophyll Concentration, LA: leaf area. Orthogonal polynomial contrasts were employed to evaluate linear (*lin*) and quadratic (*qua*) trends.

Treatment	Plant Height (cm)	Leaves (no./Plant)	Leaf Area (cm^2^/Plant)	ChlC (µmol m^−2^ LA)	Plant FW (g/Plant)	Shoot DW (g/Plant)	Root DW (g/Plant)	Plant DM (g 100 g^−1^ FW)
Substrate type (ST)	ns ^a^	*	***	***	***	***	***	*
Peat Replacement (PR)	ns	ns	***	***	***	***	*	ns
PR*lin*	ns	ns	***	*	***	***	**	ns
PR*qua*	ns	*	***	***	***	***	ns	ns
ST × PR	*	***	***	***	***	***	***	***
ST × PR*lin*	ns	***	***	***	**	ns	***	***
ST × PR*qua*	ns	*	***	**	***	**	*	ns

^a^ ns, *, **, and ***, not significant or significant at *p* ≤ 0.05, *p* ≤ 0.01, or *p* ≤ 0.001, respectively.

**Table 5:** ANOVA results showing the effect of Substrate type (ST) and peat replacement (PR) on leachate pH and EC (electrical conductivity) values in ‘Victoria’ genotype. (Data collected on 15 December 2023). Orthogonal polynomial contrasts were employed to evaluate linear (*lin*) and quadratic (*qua*) trends.

‘Victoria’	pH	EC (dS m^−1^)
Substrate type (ST)	*** ^a^	***
Peat replacement (PR)	***	***
PR*lin*	***	***
PR*qua*	***	***
ST × PR	***	***
ST × PR*lin*	***	***
ST × PR*qua*	***	*

^a^ * and ***, significant at *p* ≤ 0.05, or *p* ≤ 0.001, respectively.

**Table 6:** ANOVA results showing the effect of Substrate type (ST) and peat replacement (PR) on leachate pH and EC (electrical conductivity) values in ‘Amistad’ genotype. (Data collected on 15 December 2023). Orthogonal polynomial contrasts were employed to evaluate linear (*lin*) and quadratic (*qua*) trends.

‘Amistad’	pH	EC (dS m^−1^)
Substrate type (ST)	*** ^a^	ns
Peat replacement (PR)	***	**
PR*lin*	***	**
PR*qua*	***	**
ST × PR	ns	ns
ST × PR*lin*	ns	ns
ST × PR*qua*	ns	ns

^a^ ns, **, and ***, not significant or significant at *p* ≤ 0.01 or *p* ≤ 0.001, respectively.

**Table 7:** Substrate treatments used in the trial. The mixtures were formulated using peat as the primary organic component, with increasing levels of peat replacement (ranging from 0% to 40%) by alternative organic matrices: wood fiber (WF), coffee silverskin (CS), and brewer’s spent grain (BSG).

Substrate Treatment Code	Peat	Peat Replacement	Matrices Used as Peat Replacement
	(% *v*/*v* of the Organic Fraction of the Substrate)		
0PR	100	0	None
WF10	90	10	Wood fiber
WF20	80	20	
WF40	60	40	
CS10	90	10	Coffee silverskin
CS20	80	20	
CS40	60	40	
BSG10	90	10	Brewer’s spent grain
BSG20	80	20	
BSG40	60	40	

## Data Availability

Data are contained within the article and Supplementary Materials.

## Supplementary Materials

The following supporting information can be downloaded at: https://www.mdpi.com/article/10.3390/plants14172801/s1, Table S1: Botanical and ornamental characteristics in cv. Victoria and Amistad^®^.
