# Supplementary material for: Partial Replacement of Peat: Effects on Substrate Physico-Hydrological Properties and Sage Growth
Source: Plants (Basel). 2025 Sep 7;14(17):2801. doi: 10.3390/plants14172801 (PMC12431024; doi:10.3390/plants14172801)
Supplement: Supplementary file 1 [file plants-14-02801-s001.zip › plants-3833836-supplementary.pdf]

Table S1

Table S1. Botanical and ornamental characteristics in cv. Victoria and Amistad®.

Features	cv. Victoria	cv. Amistad®
Common Name	Victoria Blue sage	Amistad sage, Friendship sage
Life Cycle	Perennial (often grown as an annual in colder regions)	Perennial (often grown as an annual in colder regions)
Height (cm)	30–60	100–150
Growth Habit	Compact	Upright, tall, bushy
Leaves	Narrow, lance-shaped, mid-green	Several ovate, aromatic, serrated edges, dark green
Inflorescence	Dense, upright spikes of small tubular flowers	Spikes of tubular flowers
Flower Color	Intense blue-violet	Deep purple with near-black calyces
Bloom Period	Spring- Winter	Spring - Late Fall
Propagation	Seeds or stem cuttings	Primarily by cuttings (sterile hybrid)
